# Clinical Prediction of Necrotizing Pneumonia in Children With *Mycoplasma pneumoniae*: A Model Based on Early Response to Therapy

**DOI:** 10.1155/carj/3623045

**Published:** 2026-08-04

**Authors:** Jia Feng Zheng, Meng Yao Zhang, Han Quan Dong, Bo Wang, Yong Sheng Xu

**Affiliations:** ^1^ Department of Pediatric Respiratory Medicine, Children’s Hospital, Tianjin University/Tianjin Children’s Hospital, Tianjin, China, hardinge.org

## Abstract

**Objective:**

This study aimed to identify factors that predict progression to necrotizing pneumonia (NP) in children with *Mycoplasma pneumoniae* pneumonia (MPP) who fail to respond to initial therapy and to develop a predictive model to support early clinical decision‐making.

**Methods:**

We retrospectively analyzed clinical and laboratory data of children with macrolide‐unresponsive *Mycoplasma pneumoniae* pneumonia (MUMPP) admitted to Tianjin Children’s Hospital from January 2021 to January 2025. MUMPP was defined as persistent fever (≥ 38.5°C, lasting ≥ 4 h/day) after ≥ 72 h of standardized intravenous macrolide therapy. Patients were classified into an observation group (*n* = 106, those who developed NP) and a control group (*n* = 130, those who did not). Independent predictors of NP were identified by multivariable logistic regression and incorporated into a nomogram. Model performance was evaluated using ROC analysis, calibration plots, and decision curve analysis.

**Results:**

In multivariable analysis, increased white blood cell (WBC) count (OR = 2.484, 95% CI: 1.406–4.613), longer fever duration (OR = 1.618, 95% CI: 1.217–2.193), pleural effusion (OR = 3.955, 95% CI: 1.664–10.00), and elevated C‐reactive protein (CRP) (OR = 2.152, 95% CI: 1.547–3.074) were independently associated with NP among children with MPP after initial treatment failure. Calibration curves indicated close agreement between predicted and observed risks in both the derivation cohort (*p* = 0.588) and the validation cohort (*p* = 0.424). The model showed good discrimination, with an AUC of 0.848 (95% CI: 0.789–0.908) in the derivation cohort and 0.889 (95% CI: 0.815–0.963) in the validation cohort. Decision curve analysis suggested net clinical benefit across relevant threshold probabilities.

**Conclusion:**

Among children with MUMPP, higher WBC count, prolonged fever, pleural effusion, and higher CRP identify increased risk of NP.

## 1. Introduction

*Mycoplasma pneumoniae* (MP) represents one of the most prevalent pathogens responsible for community‐acquired pneumonia among pediatric populations, especially in school‐aged children (≥ 5 years) [1]. Epidemiological data indicate that the proportion of childhood community‐acquired pneumonia attributable to MP ranges from approximately 10%–40%, with an increasing trend observed in recent decades [2]. Although the majority of *Mycoplasma pneumoniae* pneumonia (MPP) cases follow a benign clinical course, a subset of approximately 12% may progress to severe *Mycoplasma pneumoniae* pneumonia (SMPP) or refractory *Mycoplasma pneumoniae* pneumonia (RMPP) [3]. Currently, macrolide antibiotics, including azithromycin and erythromycin, are universally endorsed as the preferred initial therapeutic agents for pediatric MPP [4]. However, an estimated 15%–30% of affected children fail to achieve clinical resolution within 72 h of standard macrolide administration, manifesting as ongoing or aggravated febrile episodes and respiratory symptoms—a clinical entity designated as macrolide‐unresponsive *Mycoplasma pneumoniae* pneumonia (MUMPP) by contemporary practice guidelines [4, 5].

The 72‐h time point at which MUMPP is defined represents a critical clinical decision juncture for evaluating therapeutic response and adjusting treatment regimens [6]. Early identification of children at high risk of progressing to necrotizing pneumonia (NP) enables timely interventions—such as modifying antibiotic therapy, initiating corticosteroids, or performing early pleural drainage—which may reduce the incidence of NP and adverse outcomes [7]. However, current studies addressing the progression from initial treatment failure to NP are primarily single‐center analyses with small sample sizes, lacking a unified risk assessment system [8, 9]. Existing predictive models for NP have generally been developed for all children with MPP [10] and do not specifically target the MUMPP subgroup, thereby limiting their predictive utility at the 72‐h decision point when clinicians must decide whether to escalate therapy.

To address this gap, we conducted a retrospective cohort study specifically targeting children with MUMPP, aiming to identify risk factors associated with progression to NP within this high‐risk subgroup and to establish a targeted predictive model applicable at the critical 72‐h decision point. This model is intended to provide clinicians with a practical and reliable tool for risk stratification at key therapeutic junctures, thereby supporting timely and individualized intervention strategies to improve patient outcomes.

## 2. Methods

### 2.1. Study Design and Participants

A retrospective observational design was adopted for this investigation. Eligible subjects were children admitted to the respiratory ward of Tianjin Children’s Hospital during the period from January 2021 through January 2025. Each participant had a confirmed diagnosis of MPP and satisfied the criteria for MUMPP. Based on their subsequent disease trajectory, participants were stratified into two cohorts: an observation group comprising 106 patients whose condition evolved into *Mycoplasma pneumoniae* necrotizing pneumonia (MPNP), and a control group of 130 patients who remained free of NP throughout their hospital stay and subsequent follow‐up period.

### 2.2. Definitions and Diagnostic Criteria

MPP was diagnosed in accordance with the Guidelines for the Diagnosis and Treatment of *Mycoplasma pneumoniae* Pneumonia in Children (2023 Edition) [5]. The diagnostic framework required the concurrent presence of (1) clinical respiratory manifestations such as fever, cough, wheezing, dyspnea, or auscultatory crackles; (2) radiological abnormalities compatible with MPP; and (3) microbiological confirmation through at least one of the following: a single‐sample MP antibody titer exceeding 1:160 by particle agglutination or a four‐fold or greater titer increase over the illness course; alternatively, detection of MP‐DNA or MP‐RNA from sputum or bronchoalveolar lavage specimens.

MUMPP was operationally defined as fever persisting at ≥ 38.5°C for no fewer than 4 h per day despite completion of at least 72 h of intravenous macrolide administration (azithromycin at 10 mg/kg/day for a minimum of 3 consecutive days or erythromycin at 20–30 mg/kg/day divided into 2‐3 infusions for at least 3 days), consistent with prevailing guideline recommendations [4, 5].

**FIGURE 1:**
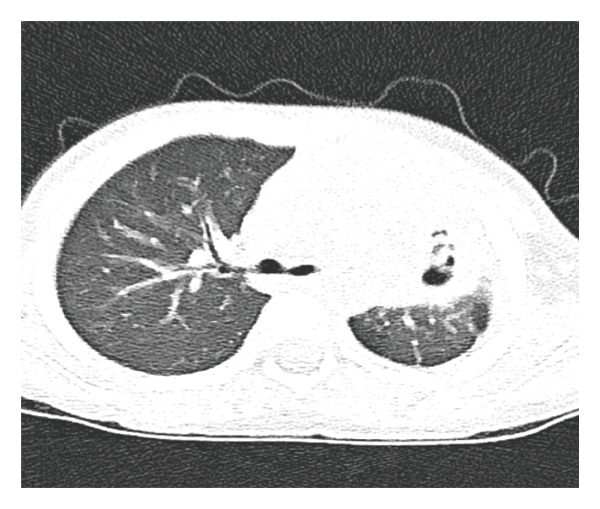
Representative axial chest CT image showing left lung consolidation with low‐attenuation foci and cavitary changes consistent with *Mycoplasma pneumoniae* necrotizing pneumonia.

The radiological diagnosis of NP was established when chest CT demonstrated multiple hypoattenuating areas, thin‐walled cavitary lesions, or cavitation lacking a discernible wall within consolidated lung regions—potentially coalescing into larger cavitary structures (Figure 1)—or when contrast‐enhanced CT revealed nonenhancing zones within areas of parenchymal consolidation [11].

### 2.3. Inclusion and Exclusion Criteria

Eligibility required fulfillment of both MPP and MUMPP diagnostic criteria together with at least one confirmatory microbiological result: serum MP antibody titer above 1:160; MP‐DNA positivity by polymerase chain reaction in bronchoalveolar lavage or pleural fluid specimens; or identification of high‐copy‐number MP sequences through targeted next‐generation sequencing (tNGS). Patients allocated to the MPNP cohort were further required to exhibit CT features consistent with NP, whereas those in the control cohort demonstrated no radiological evidence of NP at any point during their clinical course.

Exclusion criteria encompassed congenital cardiac anomalies, tuberculosis, primary or secondary immunodeficiency, connective tissue diseases, readmission during the convalescent phase of a prior pulmonary infection, congenital pulmonary malformations (including pulmonary cysts and sequestration), and lung abscess. Additionally, any patient with laboratory‐documented coinfection involving bacterial, viral, or fungal agents was excluded to reduce potential confounding effects.

### 2.4. Data Collection

Relevant clinical data were abstracted from electronic medical records in a systematic manner, encompassing demographics, symptomatology, radiological findings, and laboratory parameters. A uniform pathogen detection protocol was applied to all participants upon hospital admission; this included aerobic blood cultures, a multiplex respiratory pathogen screening panel (targeting respiratory syncytial virus, influenza A/B, parainfluenza virus types 1 through 3, adenovirus, and *Chlamydia pneumoniae*), microbiological culture of lower respiratory tract specimens, and tNGS when deemed clinically appropriate.

The imaging‐based confirmation of NP relied on official CT interpretations rendered by attending‐level radiologists. For all observation‐group cases, CT scans were re‐evaluated independently by two senior pediatric thoracic radiologists, each with over a decade of subspecialty experience. Discrepant interpretations were resolved via consensus conference or, when necessary, adjudication by an additional senior radiologist. Interpreting radiologists were masked to clinical outcome data at the time of their review.

### 2.5. Statistical Analysis

Continuous variables conforming to a normal distribution are expressed as mean ± standard deviation and compared via Student’s *t*‐test; those with skewed distributions are reported as median with interquartile range and analyzed using the Mann–Whitney *U* test. Categorical data are summarized as frequencies and percentages and compared with the chi‐square test. Two cases lacking essential outcome or predictor data were excluded from analysis. When individual variables contained sparse missing entries (below 5%), the multivariate imputation by chained equation (MICE) algorithm was applied with five iterations to generate imputed datasets.

Variable selection for the multivariable logistic regression model proceeded in two sequential stages. Initially, each of the 27 candidate predictors was evaluated via univariable logistic regression; variables reaching a significance threshold of *p* < 0.10 advanced to the next phase. These preselected covariates were subsequently incorporated into a multivariable logistic regression framework, from which predictors were removed through backward stepwise elimination guided by the Akaike Information Criterion (AIC). The parsimonious final model comprised 4 predictors, corresponding to an events‐per‐variable ratio of 26.5. Potential multicollinearity among retained predictors was examined through variance inflation factors, all of which fell below 5. A complementary sensitivity analysis that simultaneously entered all 27 variables into a single model with AIC‐based backward elimination confirmed identical predictor selection.

The predictive nomogram was generated using the “rms” package within the *R* statistical environment. Internal validation employed a random partition of the study population into a training subset (*n* = 165) and a testing subset (*n* = 71) at an approximate 7:3 ratio. Discriminative capacity was quantified by the area under the receiver operating characteristic curve (AUC), while calibration accuracy was appraised through calibration plots supported by the Hosmer–Lemeshow goodness‐of‐fit test. Clinical usefulness was further assessed via decision curve analysis. A two‐tailed *p* value below 0.05 was considered statistically significant. All computations were executed in *R* software (Version 3.4.3).

### 2.6. Ethics Statement

Ethical approval for this investigation was granted by the Institutional Review Board of Tianjin Children’s Hospital (Approval No. 2024‐LXKY‐016). Prior to analysis, all patient records underwent deidentification procedures, including the removal of names, hospital identification numbers, and any other personally identifiable information.

## 3. Results

### 3.1. Clinical Characteristics

A total of 250 pediatric patients underwent initial screening, of whom 14 were subsequently excluded for the following reasons: failure to meet inclusion criteria (*n* = 2), documented coinfections with additional pathogens (*n* = 6), presentation during the convalescent phase of MPP (*n* = 4), and incomplete medical records (*n* = 2). Consequently, 236 children with verified MPP constituted the final study population—106 who progressed to NP following unsuccessful initial treatment (observation group) and 130 who did not exhibit NP throughout their clinical course (control group).

Baseline demographics, laboratory measurements, and radiological characteristics for both groups within the full cohort (*n* = 236) are summarized in Table 1. Relative to the control group, patients in the observation group demonstrated statistically significant elevations in cough duration, fever duration, prevalence of pleural effusion, atelectasis, pleuritis, white blood cell (WBC) count, neutrophil percentage, C‐reactive protein (CRP), AST, IL‐6, and ALT (all *p* < 0.05), collectively reflecting a more pronounced disease burden. Table 2 further illustrates that the randomly allocated training and validation subsets were well‐balanced across all clinical, laboratory, and radiological parameters, thereby verifying the comparability of the two subgroups for subsequent model construction and testing.

**TABLE 1:** General clinical baseline data, imaging, and laboratory indicators of NNP and MPNP.

	NNP	MPNP	*p*
Gender			0.330
Male	52 (40.00%)	50 (47.17%)	
Female	78 (60.00%)	56 (52.83%)	
Age	9.00 [7.00; 11.00]	9.00 [7.00; 10.00]	0.167
Weight	31.75 [23.70; 43.45]	39.50 [23.38; 43.70]	0.441
Height	135.00 [115.00; 155.00]	138.00 [116.00; 152.00]	0.99
Thermal peak	38.70 [37.90; 39.30]	38.80 [38.70; 38.90]	0.149
Febrile period	8.00 [6.00; 10.00]	14.00 [9.00; 17.00]	< 0.001
Duration of cough	11.00 [9.25; 13.00]	19.00 [12.00; 24.00]	< 0.001
History of eczema			0.179
No	64 (49.23%)	42 (39.62%)	
Yes	66 (50.77%)	64 (60.38%)	
History of rhinitis			0.896
No	66 (50.77%)	52 (49.06%)	
Yes	64 (49.23%)	54 (50.94%)	
Rales	130 (100.00%)	106 (100.00%)	.
Unilateral			0.165
No	57 (43.85%)	57 (53.77%)	
Yes	73 (56.15%)	49 (46.23%)	
Atelectasis			0.007
No	119 (91.54%)	83 (78.30%)	
Yes	11 (8.46%)	23 (21.70%)	
Pleural effusion			< 0.001
No	115 (88.46%)	40 (37.74%)	
Yes	15 (11.54%)	66 (62.26%)	
Pleurisy			0.001
No	78 (60.00%)	40 (37.74%)	
Yes	52 (40.00%)	66 (62.26%)	
Breath sound low			0.132
No	56 (43.08%)	57 (53.77%)	
Yes	74 (56.92%)	49 (46.23%)	
WBC, median (Q1, Q3)	7.12 [5.55; 8.73]	15.15 [13.93; 16.50]	< 0.001
N, median (Q1, Q3)	65.95 [60.02; 71.60]	75.05 [73.32; 76.20]	< 0.001
CRP, median (Q1, Q3)	6.50 [2.50; 15.25]	15.16 [6.23; 27.77]	< 0.001
Hb, median (Q1, Q3)	127.50 [121.25; 136.00]	126.00 [118.00; 137.00]	0.374
Plt, median (Q1, Q3)	269.50 [227.25; 338.00]	298.00 [244.00; 374.25]	0.077
Pct, median (Q1, Q3)	0.12 [0.11; 0.13]	0.15 [0.07; 0.31]	0.149
La, median (Q1, Q3)	2.56 [2.20; 2.99]	2.51 [2.29; 2.81]	0.321
Pa, median (Q1, Q3)	0.11 [0.10; 0.14]	0.12 [0.10; 0.14]	0.469
LDH, median (Q1, Q3)	320.00 [287.25; 380.00]	320.50 [288.50; 380.00]	0.783
AST, median (Q1, Q3)	23.00 [20.00; 27.75]	31.50 [21.25; 46.75]	< 0.001
ALT, median (Q1, Q3)	13.00 [9.00; 17.75]	15.50 [12.25; 23.75]	< 0.001
CK, median (Q1, Q3)	82.50 [64.00; 121.50]	69.50 [41.25; 123.25]	0.013
CKMB, median (Q1, Q3)	9.00 [7.00; 12.00]	8.00 [7.00; 9.00]	0.004
IL‐6, median (Q1, Q3)	34.28 [12.55; 57.97]	61.04 [57.01; 63.54]	< 0.001
IgE, median (Q1, Q3)	139.45 [51.19; 361.60]	132.50 [62.31; 321.40]	0.927
FER, median (Q1, Q3)	256.45 [248.67; 260.90]	296.05 [144.42; 425.00]	0.491
ESR, median (Q1, Q3)	26.00 [24.00; 42.00]	33.00 [22.00; 54.00]	0.215
D‐D, median (Q1, Q3)	0.56 [0.37; 0.94]	0.43 [0.20; 1.48]	0.086
Fg, median (Q1, Q3)	4.30 [3.80; 4.72]	3.76 [3.27; 4.74]	0.028

**TABLE 2:** General information on NNP and MPNP in training and validation sets.

Variables grouped, *n* (%)	Total (*n* = 236)	Train (*n* = 165)	Test (*n* = 71)	*p*
MPNP	130 (55)	96 (58)	34 (48)	
NNP	106 (45)	69 (42)	37 (52)	
Gender				0.531
Male	102 (43)	74 (45)	28 (39)	
Female	134 (57)	91 (55)	43 (61)	
Age	9 (7, 10)	9 (7, 10)	8 (6.5, 10)	0.172
Weight	34.4 (23.53, 43.7)	34.4 (23.1, 45)	38.8 (24.1, 43)	0.834
Height	100 (96, 102)	100 (96, 102)	100 (98, 104)	0.516
Thermal peak	38.8 (38.5, 39)	38.8 (38.5, 39)	38.8 (38.6, 39)	0.493
Febrile period	9.5 (7, 14)	9 (7, 14)	10 (7, 14)	0.256
Duration of cough	13 (10, 19.25)	13 (10, 19)	13 (10, 20)	0.623
History of eczema				0.059
No	106 (45)	67 (41)	39 (55)	
Yes	130 (55)	98 (59)	32 (45)	
History of rhinitis				0.156
No	118 (50)	88 (53)	30 (42)	
Yes	118 (50)	77 (47)	41 (58)	
Rales	236 (100)	165 (100)	71 (100)	
Unilateral				0.1
No	114 (48)	86 (52)	28 (39)	
Yes	122 (52)	79 (48)	43 (61)	
Atelectasis				0.913
No	202 (86)	142 (86)	60 (85)	
Yes	34 (14)	23 (14)	11 (15)	
Pleural effusion				0.067
No	155 (66)	115 (70)	40 (56)	
Yes	81 (34)	50 (30)	31 (44)	
Pleurisy				0.156
No	118 (50)	88 (53)	30 (42)	
Yes	118 (50)	77 (47)	41 (58)	
Breath sound low				0.065
No	113 (48)	86 (52)	27 (38)	
Yes	123 (52)	79 (48)	44 (62)	
WBC, median (Q1, Q3)	10.48 (6.73, 14.83)	9.63 (6.81, 14.8)	12.8 (6.72, 15.05)	0.481
N, median (Q1, Q3)	72.4 (65.2, 75.7)	72.1 (64.6, 75.5)	73.5 (67.5, 75.9)	0.149
CRP, median (Q1, Q3)	10.93 (3.48, 23.35)	11.91 (4.46, 26.77)	7.9 (2.7, 16.94)	0.016
Hb, median (Q1, Q3)	126.5 (120, 137)	127 (120, 137)	126 (120, 136)	0.892
Plt, median (Q1, Q3)	277 (232.25, 360)	285 (233, 360)	266 (228.5, 338.5)	0.314
Pct, median (Q1, Q3)	0.12 (0.1, 0.15)	0.12 (0.1, 0.15)	0.12 (0.09, 0.17)	0.708
La, median (Q1, Q3)	2.54 (2.2, 2.89)	2.57 (2.26, 2.99)	2.51 (2.2, 2.72)	0.269
Pa, median (Q1, Q3)	0.12 (0.1, 0.14)	0.11 (0.1, 0.14)	0.12 (0.1, 0.16)	0.073
LDH, median (Q1, Q3)	320 (288, 380)	320 (278, 380)	341 (290, 380)	0.144
AST, median (Q1, Q3)	26 (20, 35)	26 (20, 37)	25 (21, 32.5)	0.669
ALT, median (Q1, Q3)	14 (10, 21)	14 (10, 21)	14 (10.5, 21)	0.894
CK, median (Q1, Q3)	80 (55, 122.75)	79 (58, 118)	82 (52, 126)	0.698
CKMB, median (Q1, Q3)	8 (7, 11)	9 (7, 11)	8 (7, 9.5)	0.237
IL.6, median (Q1, Q3)	56.06 (29.56, 63.2)	55.28 (29.07, 62.37)	60.33 (33.48, 65.25)	0.169
LgE, median (Q1, Q3)	134.45 (53.42, 340.9)	147.8 (62.27, 357.9)	119.3 (44.04, 251.3)	0.193
FER, median (Q1, Q3)	256.75 (242.4, 271.08)	256.1 (242.5, 267.4)	258.7 (241.85, 337.55)	0.514
ESR, median (Q1, Q3)	30 (22, 45)	30 (22, 45)	30 (22, 44.5)	0.538
D.D, median (Q1, Q3)	0.5 (0.33, 1.08)	0.5 (0.33, 1.08)	0.55 (0.35, 1.1)	0.927
Fg, median (Q1, Q3)	4.2 (3.59, 4.73)	4.2 (3.6, 4.75)	4.2 (3.55, 4.66)	0.589

### 3.2. Variable Selection Using Logistic Regression

The entire variable selection process was conducted solely within the training cohort (*n* = 165). Among 27 candidate predictors subjected to univariable logistic regression analysis, 7 met the prespecified retention threshold of *p* < 0.10—namely, pleural effusion, fever duration, WBC, CRP, D‐dimer, Fg, and ALT—and were subsequently entered into the multivariable model. Following AIC‐directed backward elimination, four predictors remained in the final parsimonious model (Table 3). Specifically, the adjusted multivariable analysis revealed that elevated WBC count (OR = 2.484, 95% CI: 1.406–4.613), extended fever duration (OR = 1.618, 95% CI: 1.217–2.193), presence of pleural effusion (OR = 3.955, 95% CI: 1.664–10.00), and raised CRP levels (OR = 2.152, 95% CI: 1.547–3.074) each maintained an independent association with MPNP development in children whose initial macrolide therapy had failed.

**TABLE 3:** Univariate and multivariate logistic regression screening variables were performed on MPNP risk factors.

Variables Clinical features	Univariable analysis			Multivariable analysis^∗^		
	*β*	OR (95% CI)	*p* value	*β*	OR (95% CI)	*p* value
Pleurisy	−0.036	0.964 (0.491–1.896)	0.915	—	—	
Pleural effusion	1.451	4.265 (2.070–9.309)	0.001	1.375	3.955 (1.664–10.00)	0.001
Atelectasis	−0.655	0.519 (0.221–1.178)	0.122	—	—	
Cough duration (d)	0.039	1.040 (0.777–1.392)	0.792	—	—	
Fever duration (d)	0.516	1.675 (1.321–2.158)	0.001	0.482	1.618 (1.217–2.193)	0.001
Thermal peak (°C)	0.271	1.312 (0.891–1.947)	0.172	—	—	
Demographics						
Age (years)	−0.095	0.909 (0.806–1.022)	0.115	—	—	
Gender (male)	−0.281	0.755 (0.407–1.394)	0.371	—	—	
Height (cm)	0.009	1.010 (0.988–1.033)	0.402	—	—	
Weight (kg)	0.001	1.000 (0.979–1.022)	0.991	—	—	
Laboratory parameters						
WBC (×10^9^/L)	0.819	2.267 (1.445–3.659)	0.001	0.910	2.484 (1.406–4.613)	0.001
*N* (%)	0.297	1.346 (0.865–2.122)	0.192	—	—	
CRP (mg/L)	0.852	2.344 (1.745–3.225)	0.001	0.766	2.152 (1.547–3.074)	0.001
ESR (mm/h)	0.298	1.347 (0.849–2.158)	0.208	—	—	
PCT (ng/mL)	−0.146	0.864 (0.392–1.874)	0.716	—	—	
IL‐6 (pg/mL)	0.231	1.260 (0.905–1.766)	0.173	—	—	
D‐dimer (mg/L)	0.805	2.236 (1.465–3.523)	0.001	—	—	
Fg (g/L)	0.958	2.606 (1.145–6.772)	0.032	—	—	
FER (ng/mL)	0.101	1.106 (0.694–1.782)	0.672	—	—	
IgE (IU/mL)	−0.178	0.837 (0.583–1.191)	0.325	—	—	
Plt (×10^9^/L)	0.431	1.537 (0.610–4.094)	0.371	—	—	
ALT (U/L)	0.998	2.714 (1.106–10.10)	0.061	—	—	
AST (U/L)	0.348	1.417 (0.602–3.462)	0.431	—	—	
LDH (U/L)	−0.351	0.704 (0.335–1.466)	0.349	—	—	
Lactate (mmol/L)	−0.355	0.701 (0.433–1.124)	0.143	—	—	
CK‐MB (U/L)	0.679	1.972 (0.813–5.137)	0.144	—	—	
CK (U/L)	−0.132	0.876 (0.608–1.253)	0.469	—	—	

*Note:* The final model was determined by backward stepwise selection based on the Akaike information criterion (AIC). A *p* value < 0.05 was considered statistically significant. Dashes indicate variables not included in the multivariable model.

^∗^Variables with *p* < 0.10 in univariable analysis were entered into a multivariable logistic regression model (7 variables: pleural effusion, fever duration, CRP, WBC, D‐dimer, Fg, and ALT).

### 3.3. Development of the Clinical Prediction Model

**FIGURE 2:**
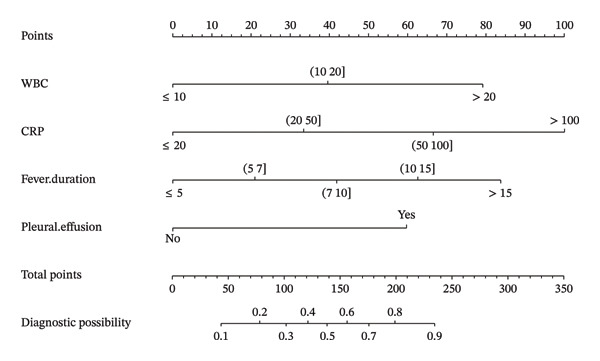
Nomogram for predicting necrotizing pneumonia (NP) in children with macrolide‐unresponsive *Mycoplasma pneumoniae* pneumonia after initial treatment failure, incorporating WBC count, CRP, fever duration, and pleural effusion.

Based on the four independently predictive variables (WBC count, fever duration, pleural effusion, and CRP), a nomogram was constructed. Within this scoring system, each predictor is assigned a corresponding point value; the aggregate of all individual scores maps onto an estimated probability of MPNP occurrence after initial therapeutic failure (Figure 2).

### 3.4. Validation of Model Calibration and Discrimination

**FIGURE 3:**
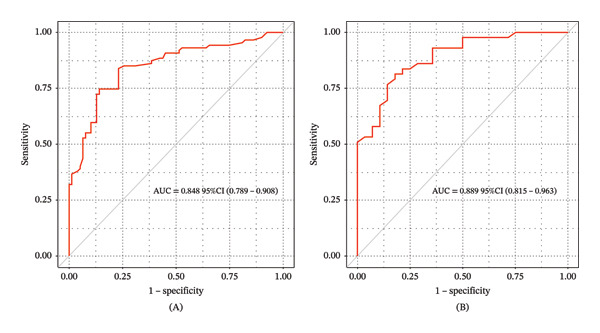
ROC curves for the prediction model. (A) Training cohort: AUC = 0.848 (95% CI: 0.789–0.908). (B) Validation cohort: AUC = 0.889 (95% CI: 0.815–0.963).

**FIGURE 4:**
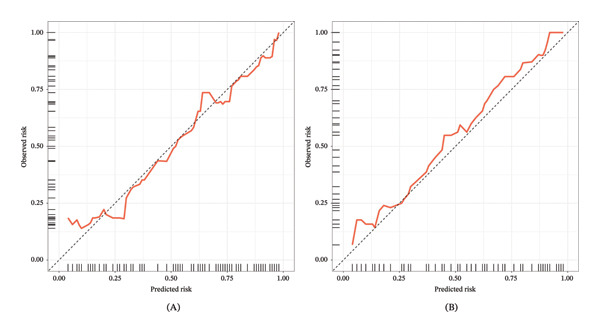
Calibration curves for the prediction model. (A) Training cohort: Hosmer–Lemeshow test *p* = 0.588. (B) Validation cohort: Hosmer–Lemeshow test *p* = 0.424.

The study cohort was partitioned at a 7:3 ratio into a derivation subset (*n* = 165) and an independent testing subset (*n* = 71). Both discriminative performance and calibration accuracy were appraised through ROC analysis and calibration curve plotting. The model achieved an AUC of 0.848 (95% CI: 0.789–0.908) in the derivation subset and 0.889 (95% CI 0.815–0.963) in the testing subset (Figure 3A,B), reflecting robust discriminative capacity. Hosmer–Lemeshow goodness‐of‐fit testing revealed no statistically significant discrepancies between model‐estimated and actually observed NP rates in either subset (derivation: *p* = 0.588; testing: *p* = 0.424) (Figure 4A,B), confirming satisfactory calibration performance.

### 3.5. Evaluation of Clinical Utility

**FIGURE 5:**
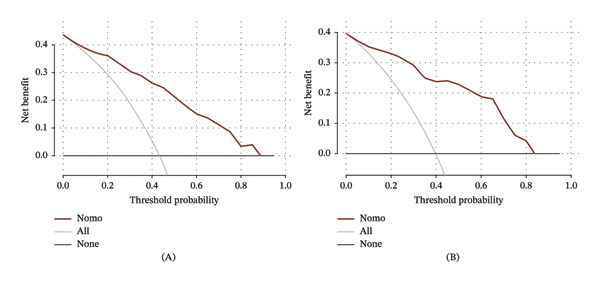
Decision curve analysis for the prediction model. (A) Training cohort: positive net benefit over thresholds of approximately 0.0820130.89. (B) Validation cohort: positive net benefit over thresholds of approximately 0.08–0.85.

Decision curve analysis demonstrated that applying the nomogram for clinical decision‐making yielded a positive net benefit over threshold probability ranges of 0.08–0.89 in the derivation subset and 0.08–0.85 in the testing subset. Across these clinically relevant probability intervals, utilizing model‐derived risk estimates can assist in identifying pediatric patients with a heightened likelihood of developing NP after initial therapeutic failure, thereby facilitating earlier institution of targeted management strategies to optimize prognosis (Figure 5A,B).

## 4. Discussion

In this retrospective study of 236 children with MUMPP, we identified four independent predictors of progression to NP: elevated WBC count (OR = 2.484, 95% CI: 1.406–4.613), prolonged fever duration (OR = 1.618, 95% CI: 1.217–2.193), presence of pleural effusion (OR = 3.955, 95% CI: 1.664–10.00), and elevated CRP (OR = 2.152, 95% CI: 1.547–3.074). The nomogram incorporating these variables demonstrated strong discriminative ability, with an AUC of 0.848 (95% CI: 0.789–0.908) in the training cohort and 0.889 (95% CI: 0.815–0.963) in the validation cohort and satisfactory calibration as indicated by nonsignificant Hosmer–Lemeshow tests (*p* = 0.588 and *p* = 0.424, respectively). Decision curve analysis confirmed net clinical benefit across threshold probabilities of approximately 0.08–0.89, supporting the model’s practical utility for guiding treatment escalation decisions. A key design feature of the present study is that enrollment was restricted to children who had already met the MUMPP definition—the critical 72‐h juncture at which macrolide therapy has demonstrably failed. Unlike prior predictive models developed for all children with MPP [10] or refractory MPP without a uniform temporal anchor [8, 9], our approach isolates the incremental risk factors for NP progression within a clinically homogeneous, high‐risk population, thereby enhancing the specificity and clinical applicability of the model precisely at the point when clinicians must decide whether to escalate therapy.

Leukocytosis is commonly observed in children with MPP who progress to NP and likely reflects an exaggerated systemic inflammatory response driven by the interplay between MP infection and host immunity. MP can activate a robust innate immune response through toll‐like receptor signaling and community‐acquired respiratory distress syndrome (CARDS) toxin–mediated pathways, leading to excessive recruitment of neutrophils and macrophages to the pulmonary parenchyma, which in turn contributes to tissue necrosis and cavitation [12]. In the context of MUMPP, persistent infection despite macrolide therapy may further amplify this inflammatory cascade, resulting in sustained or rising WBC counts that serve as a clinical marker of disease escalation. A previous study of children with NP reported that elevations in WBC, CRP, and PCT were among the most prominent inflammatory features, and that these markers were significantly higher in NP than in non‐NP [13]. Importantly, leukocytosis not only reflects the current severity of pulmonary involvement but also provides prognostic information: higher WBC counts have been associated with longer hospitalization, more complex treatment regimens, and an increased need for invasive procedures such as pleural drainage or surgical debridement [14]. Our findings are consistent with this evidence. Among children with MPP who failed initial macrolide therapy, elevated WBC count was independently associated with progression to NP, underscoring the importance of close monitoring of inflammatory markers at the 72‐h therapeutic decision point. When WBC counts remain persistently elevated or continue to rise despite treatment adjustment, clinicians should maintain a high index of suspicion for NP development and consider timely escalation of anti‐inflammatory and antimicrobial interventions to prevent further parenchymal destruction.

CRP is another important inflammatory biomarker with high sensitivity for differentiating NP. In pediatric NP, CRP levels are significantly higher than in non‐NP, and a threshold of > 48.35 mg/L has been reported to predict pulmonary necrosis, with an AUC of 0.836 on ROC analysis, indicating substantial diagnostic value [15]. CRP levels also correlate with NP severity. In bacterial NP, CRP can help distinguish infections caused by different pathogens, for example, differentiating MP from typical bacterial infections, and demonstrates good clinical utility for assessing disease severity [16]. Furthermore, CRP can guide treatment decisions and prognostic assessment. Composite indices incorporating CRP, such as the CRP‐to‐albumin ratio, have been proposed as more reliable indicators of pneumonia risk and severity and may provide a more nuanced approach to NP management [13]. Nevertheless, CRP should not be interpreted in isolation. Its diagnostic and prognostic performance is limited when used alone for severe pneumonia; its predictive value is enhanced when combined with body temperature, neutrophil count, and clinical findings [14]. In our study, we selected variables with high diagnostic value, including CRP, and integrated them with fever duration and other laboratory parameters in the predictive model, thereby improving the accuracy of NP risk estimation.

NP leads to destruction of lung parenchyma with subsequent cavitation and is often accompanied by pleural effusion. Depending on the infectious process and the presence of air in the pleural space, effusions may be classified as parapneumonic effusion, empyema, or pyopneumothorax [17]. NP is a severe complication of pediatric pneumonia characterized by tissue necrosis, cavitary lesions, and liquefaction. Pleural effusion is a common manifestation of NP. NP is most often associated with bacterial pathogens such as *Streptococcus pneumoniae* and *Staphylococcus aureus*, including methicillin‐resistant *S. aureus* (MRSA). These organisms can drive extensive parenchymal necrosis and liquefaction within cavitary lesions, thereby aggravating pleural effusion [17, 18]. MP has also been associated with NP [17, 19]. In severe MPP, extensive consolidation and atelectasis may occur, with secondary pleural effusion, findings that are consistent with our results. Clinically, fever persisting beyond 3 days suggests suboptimal infection control, and without timely intervention, the risk of developing pleural effusion increases substantially.

In pediatric pulmonary infections, fever duration is not only a common symptom but also a potential predictor of NP severity and progression. Several studies have reported that prolonged fever is associated with an increased risk of NP. One study identified fever duration > 11.5 days as a strong predictor of NP, with an AUC of 0.909 on ROC analysis, indicating high sensitivity [15]. In children with MPP, fever duration has been recognized as a key factor influencing disease course. A predictive model for NP in this setting incorporated fever duration as a central component, underscoring its importance in clinical assessment [20]. Prolonged fever in NP is frequently associated with more severe clinical outcomes, longer hospital stays, and more complex treatment regimens. One study reported a median fever duration of 13 days in children with NP, which was associated with extended hospitalization and more intensive interventions (NP in children). Persistent fever may also reflect more severe NP, higher mortality, and increased need for surgical procedures [21]. Fever duration is thus not only a symptom but also an important diagnostic and prognostic tool. Logistic regression analysis in one study demonstrated that fever duration, combined with inflammatory markers, could predict the likelihood of NP [22]. Our study similarly showed that, after initial treatment failure, active anti‐infective therapy, close monitoring of inflammatory markers, and timely evaluation at key points in disease progression are crucial in reducing the risk of NP. By incorporating fever duration (≥ 3 days) together with laboratory indices such as WBC and CRP, our predictive model facilitates early identification of children at high risk for NP and prompts clinicians to implement more proactive clinical interventions.

In developing the multivariable prediction model, we adopted a two‐stage approach univariable prescreening followed by AIC‐guided backward stepwise selection to balance model parsimony and clinical interpretability while ensuring an adequate events‐per‐variable ratio. Several variables that were significant in univariable analysis, including D‐dimer, fibrinogen, and ALT, were eliminated during the stepwise procedure, suggesting that their predictive information was largely captured by the four retained variables. Notably, IgE did not meet the prescreening threshold (*p* = 0.325 in univariable analysis) and was not entered into the multivariable model. From a pathophysiological standpoint, IgE predominantly mediates Type I hypersensitivity and allergic responses, and its role in the vascular thrombosis, ischemic necrosis, and intense neutrophilic inflammation that underlie MP NP has not been established in the literature. Therefore, both the statistical evidence and the biological plausibility support the exclusion of IgE from the final prediction model. In a supplementary multivariable model that included all 27 candidate variables simultaneously, IgE showed borderline significance (OR = 0.560, 95% CI: 0.310–0.969, *p* = 0.044); however, it was eliminated during stepwise selection, and the severely low EPV ratio (≈3.9) of the full model renders this estimate unreliable. The consistent exclusion of IgE across both analytic approaches further supports its omission from the final prediction model.

This study has several limitations. First, despite systematic pathogen screening, undetected coinfections with low‐virulence or subclinical organisms may have contributed to inflammatory marker elevation in some patients. Second, as a retrospective, single‐center study with a limited sample size, the model was only internally validated using a random 7:3 split, which may overestimate predictive performance. Therefore, the generalizability of this nomogram to other institutions or populations remains to be established, and prospective, multicenter external validation is warranted before clinical implementation.

## 5. Conclusions

In summary, among children with MUMPP, pleural effusion, prolonged fever duration, elevated WBC count, and elevated CRP are major high‐risk factors for progression to NP. The clinical prediction model constructed from these variables provides a simple and evidence‐based tool for estimating NP risk, enabling clinicians to intervene early in the course of NP development and progression and thereby improving clinical outcomes. However, as this model was only internally validated, prospective multicenter external validation is essential before clinical application.

**Nomenclature**
NNP: Non‐necrotizing pneumonia
WBC: White blood cell
N: Neutrophil
CRP: C‐reactive protein
Hb: Hemoglobin
Plt: Platelet
Pct: Procalcitonin
La: Lactate
PA: Prealbumin
LDH: Lactate dehydrogenase
AST: Aspartate aminotransferase
ALT: Alanine aminotransferase
CK: Creatine kinase
CKMB: Creatine kinase‐MB
IL‐6: Interleukin‐6
IgE: Immunoglobulin E
FER: Ferritin
D‐D: D‐dimer
ESR: Erythrocyte sedimentation rate
Fg: Fibrinogen
MPNP: *Mycoplasma pneumoniae* necrotizing pneumonia
MPP: *Mycoplasma pneumoniae* pneumonia
RMPP: Refractory *Mycoplasma pneumoniae* pneumonia
SMPP: Severe *Mycoplasma pneumoniae* pneumonia
ROC: Receiver operating characteristic
PVL: Panton–valentine leukocidin
NP: Necrotizing pneumonia
MP: *Mycoplasma pneumoniae*
DCA: Decision curve analysis
AUC: Area under the curve
tNGS: Targeted next‐generation sequencing
MRSA: Methicillin‐resistant *Staphylococcus aureus*
PCR: Polymerase chain reaction
MUMPP: Macrolide‐unresponsive *Mycoplasma pneumoniae* pneumonia

## Author Contributions

Jia Feng Zheng designed the work and wrote the manuscript. Jia Feng Zheng and Meng Yao Zhang analyze the data and visualize the picture. Jia Feng Zheng, Meng Yao Zhang, Bo Wang, and Yong Sheng Xu revised the manuscript. Han Quan Dong is responsible for managing and coordinating the planning and execution of research activities. Jia Feng Zheng and Meng Yao Zhang were the main contributors to the writing of the manuscript.

## Funding

This study was sponsored by Tianjin Health Research Project (Grant no.TJWJ2024MS035), funded by Tianjin Key Medical Discipline Construction Project (Grant no.TJYXZDXK‐3‐016B).

## Disclosure

All authors have read and approved the final manuscript.

## Ethics Statement

This study was approved by the Ethics Committee of Tianjin Children’s Hospital (Approval No. 2024‐LXKY‐016). Written informed consent was obtained from the guardians of all participating children.

## Consent

Please see the Ethics Statement.

## Conflicts of Interest

The authors declare no conflicts of interest.

## Data Availability

The data that support the findings of this study are available from the corresponding authors upon reasonable request.
